# Supplementary material for: Habitat complexity and benthic predator-prey interactions in Chesapeake Bay
Source: PLoS One. 2018 Oct 5;13(10):e0205162. doi: 10.1371/journal.pone.0205162 (PMC6173400; doi:10.1371/journal.pone.0205162)
Supplement: S1 Table — For each pairwise comparison, 95% confidence intervals (CI) and adjusted p values are presented. Data were Box-Cox transformed (λ = 0.51) prior to analysis and are not back-transformed. Only interactions with significant p values at α = 0.20 are shown. (PDF) [file pone.0205162.s001.pdf]

S1 Table. Summary of Tukey HSD results for the caging study interaction term between habitat and cage type. For each pairwise comparison, 95% confidence intervals (CI) and adjusted p values are presented. Data were Box-Cox transformed ($\lambda = 0.51$) prior to analysis and are not back-transformed. Only interactions with significant p values at $\alpha = 0.20$ are shown.

Habitat and Cage Type Comparison	*Difference*	*Lower CI*	*Upper CI*	*Adjusted p value*
Mud x Stockade-Mud x Full	-1.56	-2.08	-1.03	< 1.0E-7
Sand x Stockade-Mud x Full	-0.97	-1.50	-0.45	3.1E-06
Seagrass x Stockade-Mud x Full	-0.66	-1.18	-0.13	0.004
Mud x Uncaged-Mud x Full	-1.34	-1.87	-0.82	< 1.0E-7
Sand x Uncaged-Mud x Full	-0.77	-1.30	-0.25	0.0004
Seagrass x Uncaged-Mud x Full	-1.07	-1.60	-0.55	2E-07
Mud x Stockade-Sand x Full	-1.53	-2.09	-0.96	< 1.0E-7
Sand x Stockade-Sand x Full	-0.94	-1.51	-0.38	3.09E-05
Seagrass x Stockade-Sand x Full	-0.63	-1.19	-0.06	0.02
Mud x Uncaged-Sand x Full	-1.31	-1.87	-0.75	< 1.0E-7
Sand x Uncaged-Sand x Full	-0.74	-1.30	-0.18	0.002
Seagrass x Uncaged-Sand x Full	-1.04	-1.61	-0.48	3.1E-06
Mud x Stockade-Seagrass x Full	-1.61	-2.15	-1.07	< 1.0E-7
Sand x Stockade-Seagrass x Full	-1.03	-1.57	-0.49	1.7E-06
Seagrass x Stockade-Seagrass x Full	-0.71	-1.26	-0.17	0.002
Mud x Uncaged-Seagrass x Full	-1.40	-1.94	-0.85	< 1.0E-7
Sand x Uncaged-Seagrass x Full	-0.83	-1.37	-0.29	0.0002
Seagrass x Uncaged-Seagrass x Full	-1.13	-1.67	-0.59	1E-07
Sand x Stockade-Mud x Stockade	0.58	0.07	1.09	0.01
Seagrass x Stockade-Mud x Stockade	0.90	0.39	1.41	1.06E-05
Sand x Uncaged-Mud x Stockade	0.79	0.27	1.30	0.0002
Seagrass x Uncaged-Mud x Stockade	0.48	-0.03	0.99	0.08
Mud x Uncaged-Seagrass x Stockade	-0.68	-1.19	-0.17	0.002
Seagrass x Uncaged-Seagrass x Stockade	-0.42	-0.93	0.09	0.20
Sand x Uncaged-Mud x Uncaged	0.57	0.06	1.08	0.02
