# Supplementary material for: Habitat complexity and benthic predator-prey interactions in Chesapeake Bay
Source: PLoS One. 2018 Oct 5;13(10):e0205162. doi: 10.1371/journal.pone.0205162 (PMC6173400; doi:10.1371/journal.pone.0205162)
Supplement: S2 Table — For each pairwise comparison, 95% confidence intervals (CI) and adjusted p values are presented. Data were Box-Cox transformed (λ = -0.14) prior to analysis and are not back-transformed. Only interactions with significant p values at α = 0.20 are shown. (PDF) [file pone.0205162.s002.pdf]

S2 Table. Summary of Tukey HSD results for the mesocosm study proportional mortality interaction term between species and density. For each pairwise comparison, 95% confidence intervals (CI) and adjusted p values are presented. Data were Box-Cox transformed ($\lambda = -0.14$) prior to analysis and are not back-transformed. Only interactions with significant p values at $\alpha = 0.20$ are shown.

Species and Density Comparison	*Difference*	*Lower CI*	*Upper CI*	*Adjusted p value*
Mya x medium-Mya x low	-0.34	-0.62	-0.07	0.01
Mya x medium-Mercenaria x low	-0.44	-0.71	-0.16	0.0004
Mya x medium-Mercenaria x medium	-0.49	-0.22	-0.77	0.00005
