# Supplementary material for: Habitat complexity and benthic predator-prey interactions in Chesapeake Bay
Source: PLoS One. 2018 Oct 5;13(10):e0205162. doi: 10.1371/journal.pone.0205162 (PMC6173400; doi:10.1371/journal.pone.0205162)
Supplement: S3 Table — For each pairwise comparison, 95% confidence intervals (CI) and adjusted p values are presented. Data were Box-Cox transformed (λ = -0.14) prior to analysis and are not back-transformed. Only interactions with significant p values at α = 0.20 are shown. (PDF) [file pone.0205162.s003.pdf]

S3 Table. Summary of Tukey HSD results for the mesocosm study bivalve proportional mortality interaction term between species and habitat. For each pairwise comparison, 95% confidence intervals (CI) and adjusted p values are presented. Data were Box-Cox transformed (λ = -0.14) prior to analysis and are not back-transformed. Only interactions with significant p values at α = 0.20 are shown.

Species and Habitat Comparison	*Difference*	*Lower CI*	*Upper CI*	*Adjusted p value*
Mya x sand-Mercenaria x oyster	-0.57	-1.03	-0.11	0.01
Mya x sand-Mercenaria x sand	-0.47	-0.93	-0.01	0.04
Mya x sand-Mercenaria x seagrass	-0.64	-1.10	-0.18	0.001
Mya x seagrass-Mercenaria x seagrass	-0.48	-0.94	-0.02	0.04
Mya x seagrass-Mercenaria x oyster	-0.41	-0.87	0.05	0.12
