# Supplementary material for: Habitat complexity and benthic predator-prey interactions in Chesapeake Bay
Source: PLoS One. 2018 Oct 5;13(10):e0205162. doi: 10.1371/journal.pone.0205162 (PMC6173400; doi:10.1371/journal.pone.0205162)
Supplement: S4 Table — For each pairwise comparison, 95% confidence intervals (CI) and adjusted p values are presented. Data were fourth-root transformed prior to analysis and are not back-transformed. Only interactions with significant p values at α = 0.20 are shown. (PDF) [file pone.0205162.s004.pdf]

S4 Table. Summary of Tukey HSD results for the mesocosm study *Callinectes sapidus* handling time interaction term between species and habitat. For each pairwise comparison, 95% confidence intervals (CI) and adjusted p values are presented. Data were fourth-root transformed prior to analysis and are not back-transformed. Only interactions with significant p values at α = 0.20 are shown.

Species and Habitat Comparison	*Difference*	*Lower CI*	*Upper CI*	*Adjusted p value*
Mercenaria x shell-Mya x oyster	0.54	-0.02	1.1	0.06
Mercenaria x shell-Mya x shell	0.47	-0.09	1.02	0.15
Mercenaria x shell-Mercenaria x sand	0.46	-0.1	1.02	0.17
