# Supplementary material for: Habitat complexity and benthic predator-prey interactions in Chesapeake Bay
Source: PLoS One. 2018 Oct 5;13(10):e0205162. doi: 10.1371/journal.pone.0205162 (PMC6173400; doi:10.1371/journal.pone.0205162)
Supplement: S5 Table — For each pairwise comparison, 95% confidence intervals (CI) and adjusted p values are presented. Data were fourth-root transformed prior to analysis and are not back-transformed. Only interactions with significant p values at α = 0.20 are shown. (PDF) [file pone.0205162.s005.pdf]

S5 Table. Summary of Tukey HSD results for the mesocosm study *Callinectes sapidus* search time interaction term between species and density. For each pairwise comparison, 95% confidence intervals (CI) and adjusted p values are presented. Data were fourth-root transformed prior to analysis and are not back-transformed. Only interactions with significant p values at α = 0.20 are shown.

Species and Density Comparison	*Difference*	*Lower CI*	*Upper CI*	*Adjusted p value*
Mya x med-Mya x low	0.57	0.24	0.91	0.0003
Mya x med-Mercenaria x low	0.35	0.02	0.69	0.04
Mercenaria x med-Mya x med	-0.37	-0.70	-0.03	0.03
