# Supplementary material for: Habitat complexity and benthic predator-prey interactions in Chesapeake Bay
Source: PLoS One. 2018 Oct 5;13(10):e0205162. doi: 10.1371/journal.pone.0205162 (PMC6173400; doi:10.1371/journal.pone.0205162)
Supplement: S6 Table — For each pairwise comparison, 95% confidence intervals (CI) and adjusted p values are presented. Data were fourth-root transformed prior to analysis and are not back-transformed. Only interactions with significant p values at α = 0.20 are shown. (PDF) [file pone.0205162.s006.pdf]

S6 Table. Summary of Tukey HSD results for the mesocosm study *Callinectes sapidus* search time interaction term between species, density, and habitat. For each pairwise comparison, 95% confidence intervals (CI) and adjusted p values are presented. Data were fourth-root transformed prior to analysis and are not back-transformed. Only interactions with significant p values at α = 0.20 are shown.

Species, Density, and Habitat Comparison	*Difference*	*Lower CI*	*Upper CI*	*Adjusted p value*
shell x Mercenaria x low-oyster x Mya x low	0.77	-0.15	1.69	0.18
oyster x Mya x med-oyster x Mya x low	1.04	0.12	1.96	0.02
sand x Mya x med-oyster x Mya x low	0.96	0.04	1.88	0.03
seagrass x Mya x med-oyster x Mya x low	1.06	0.14	1.98	0.01
